# Decompressive Craniectomy for Traumatic Brain Injury: Postoperative TCD Cerebral Hemodynamic Evaluation

**DOI:** 10.3389/fneur.2019.00354

**Published:** 2019-04-12

**Authors:** Edson Bor-Seng-Shu, Marcelo de-Lima-Oliveira, Ricardo Carvalho Nogueira, Kelson James Almeida, Eric Homero Albuquerque Paschoal, Fernando Mendes Paschoal

**Affiliations:** ^1^Laboratory for Neurosonology and Cerebral Hemodynamics, Division of Neurological Surgery, Hospital das Clinicas, São Paulo University Medical School, São Paulo, Brazil; ^2^Department of Neurology, Federal University of Piauí Medical School, Teresina, Brazil; ^3^Department of Neurology, Federal University of Pará Medical School, São Paulo, Brazil

**Keywords:** decompressive craniectomy, traumatic brain injury, transcranial Doppler ultrasonography, intracranial pressure (ICP), cerebral hemodynamics

## Abstract

**Background:** There are no studies describing the cerebral hemodynamic patterns that can occur in traumatic brain injury (TBI) patients following decompressive craniectomy (DC). Such data have potentially clinical importance for guiding the treatment. The objective of this study was to investigate the postoperative cerebral hemodynamic patterns, using transcranial Doppler (TCD) ultrasonography, in patients who underwent DC. The relationship between the cerebral circulatory patterns and the patients' outcome was also analyzed.

**Methods:** Nineteen TBI patients with uncontrolled brain swelling were prospectively studied. Cerebral blood circulation was evaluated by TCD ultrasonography. Patients and their cerebral hemispheres were categorized based on TCD-hemodynamic patterns. The data were correlated with neurological status, midline shift on CT scan, and Glasgow outcome scale scores at 6 months after injury.

**Results:** Different cerebral hemodynamic patterns were observed. One patient (5.3%) presented with cerebral oligoemia, 4 patients (21%) with cerebral hyperemia, and 3 patients (15.8%) with cerebral vasospasm. One patient (5.3%) had hyperemia in one cerebral hemisphere and vasospasm in the other hemisphere. Ten patients (52.6%) had nonspecific circulatory pattern. Abnormal TCD-circulatory patterns were found in 9 patients (47.4%). There was no association between TCD-cerebral hemodynamic findings and outcome.

**Conclusion:** There is a wide heterogeneity of postoperative cerebral hemodynamic findings among TBI patients who underwent DC, including hemodynamic heterogeneity between their cerebral hemispheres. DC was proved to be effective for the treatment of cerebral oligoemia. Our data support the concept of heterogeneous nature of the pathophysiology of the TBI and suggest that DC as the sole treatment modality is insufficient.

## Introduction

Decompressive craniectomy (DC) may effectively decrease intracranial pressure (ICP) and increase cerebral perfusion pressure (CPP) in traumatic brain injury (TBI) patients with refractory elevated ICP (1, 2). However, randomized controlled trials failed to disclose the efficacy of this procedure for improving these patients' neurological outcome (3, 4). This means that ICP control to ensure CPP, as the sole treatment strategy, is not sufficient to achieve satisfactory therapeutic results in most cases. New research should focus on different pathophysiological mechanisms of posttraumatic brain swelling.

Transcranial Doppler (TCD) ultrasonography is a non-invasive and bedside method for real-time assessment of cerebral blood circulation. This technique is routinely used in clinical and scientific scenario (5–8).

To date, few studies have addressed the cerebral hemodynamic and metabolic effects of DC for uncontrolled elevation of ICP (2, 9–15). To our knowledge, there are no studies describing the different cerebral hemodynamic patterns that can occur in TBI patients following DC. Such data potentially have clinical importance, which justifies a study.

The aim of this study was to investigate the postoperative cerebral hemodynamic patterns, using TCD ultrasonography, in patients who underwent DC for uncontrolled intracranial hypertension and brain herniation syndrome. The relationship between the cerebral circulatory patterns and the patients' outcome was also evaluated.

## Materials and Methods

### Study Design and Patient Enrollment

A prospective study on the effects of DC on cerebral hemodynamics for traumatic brain injury (TBI) was conducted from January 1999 to September 2002, at the Hospital das Clinicas of the University of Sao Paulo Medical School. The enrollment criteria were TBI patients presenting with severe brain swelling for whom DC was indicated and in whom preoperative and postoperative TCD ultrasonography had been carried out. Exclusion criteria included penetrating TBI, Glasgow Coma Scale (GCS) score of 3 associated with bilaterally fixed and dilated pupils, and the lack of TCD ultrasonography evaluations. Multisystem trauma patients were not excluded. Participants were characterized in terms of demographic, clinical, and radiological variables. This study was approved by our research ethics committee (CAPPesq).

### Patient Population

Nineteen patients met the inclusion criteria for this study. Their ages ranged from 17 to 63 years, with a mean age of 33 ± 14 years. There were 13 male and 6 female patients. Median admission GCS scores was 7, varying from 4 to 13. These patients were divided into two groups. The first group consisted of 9 patients with no focal lesions, in whom severe brain swelling and refractory signs of brain herniation led to DC. The second group was composed of 10 patients who presented with an expanding hematoma (contusion hemorrhage, extradural and/or subdural hematoma), which had been initially removed, and developed afterwards severe brain swelling. Twenty percent of patients had a hypotensive insult at hospital admission. Demographic, clinical, and imaging data for each patient were presented in our previous studies (10, 16).

### General Management Protocol

Guidelines of the American College of Surgeons (Advanced Trauma Life Support) and of the American Association of Neurological Surgeons were adopted for the clinical e surgical management of the patients (10). All patients with neurological deterioration underwent brain computerized tomography (CT) scans. ICP monitoring was not part of the study.

### Indications for Surgical Decompression

DC was performed in patients with neurological deterioration and CT scans disclosing predominantly unilateral diffuse brain swelling associated with mass effect, a midline shift and/or obliteration of peri-mesencephalic cisterns. Neurological worsening was defined as a decrease in GCS score and/or unilaterally or bilaterally unresponsive and dilated pupils. Patients with persisting GCS score of 3 and/or bilaterally fixed and dilated pupils were not operated on. The surgical technique consisted of large hemicraniectomy with dural opening over the most swollen cerebral hemisphere.

### Evaluation of Cerebral Hemodynamics

TCD examinations were performed before surgery while the patient waited to go to the operating room, or while the surgical team prepared the patients in the operating room. Postoperative TCD examinations were obtained soon after the completion of incision closure and dressing, while the anesthesiologist prepared the patient in the operating room. Portable 2 MHz pulsed TCD equipment (Pioneer TC 2020 EME; Nicolet Biomedical, Madison, WI) was used to measure the blood velocities in the middle cerebral arteries (MCA) and the distal segment of the extracranial internal carotid arteries (ICA), respectively, via temporal and submandibular regions. TCD examinations were performed by an experienced sonographer (EBSS) using a hand-held technique. Monitoring of cerebral blood flow velocities for long periods of time was not performed. The TCD variables were the mean velocity (the time mean of the peak velocities over the course of four cardiac cycles) and the pulsatility index (PI = [systolic velocity–diastolic velocity]/mean velocity).

Systemic arterial blood pressure, body temperature, hematocrit, arterial blood carbon dioxide, and oxygen pressures were noted in each TCD examination.

### Definition of TCD Cerebral Circulatory Patterns

High MCA mean blood velocities can occur in both cerebral vasospasm and hyperemia. The Lindegaard ratio (LR), defined as the ratio of MCA mean blood velocity to the ipsilateral extracranial ICA mean blood velocity, can be used to discriminate cerebral vasospasm from hyperemia (17–19). For the calculation of the LR, MCA flow velocities were divided by the ipsilateral extracranial ICA flow velocities. LR can improve the diagnostic accuracy of TCD in detecting cerebral vasospasm.

MCA mean blood velocities >100 cm/s along with LR <3 were considered as cerebral hyperemia, whereas MCA mean blood velocities <40 cm/s were defined as cerebral oligoemia. MCA mean blood velocities between 40 and 100 cm/s associated with LR <3 were considered as non-specific hemodynamic pattern. MCA mean blood velocities >100 cm/s in conjunction with LR >3 were considered as cerebral vasospasm (17–19).

### Categorization of Patients by Cerebral Circulatory Patterns

Participants with hyperemia in both cerebral hemispheres, or hyperemia in one cerebral hemisphere and non-specific hemodynamic pattern in the contralateral hemisphere were defined as having cerebral hyperemia. In contrast, participants with oligoemia in both cerebral hemispheres, or oligoemia in one cerebral hemisphere and non-specific hemodynamic pattern in another hemisphere were considered as presenting cerebral oligoemia. Patients with hyperemia in one cerebral hemisphere and oligoemia in the contralateral hemisphere, as well as patients with cerebral vasospasm, were grouped separately.

### Data Collection

Clinical data such as age, gender, accident date, time intervals from accident to hospital admission, and from admission to decompressive craniectomy, brain injury mechanisms, neurological examination (GCS score and pupil activity) at admission, prior to, and following brain decompression, midline brain structures shift, associated intracranial posttraumatic lesions, length of hospital stay, and outcome were extracted from our database.

### Clinical Outcome

Glasgow Outcome Scale (GOS) score was determined for all patients approximately 6 months post-injury. Patients were assigned to one of the five categories: death, persistent vegetative state, severe disability, moderate disability, or good recovery. Patients with good recovery (GOS score of 5) or with moderate disability (GOS score of 4) were defined as presenting favorable outcome. Patients who were assigned to the severe disability (GOS score of 3) or to the persistent vegetative state (GOS score of 2) or those who died (GOS score of 1) were considered to have an unfavorable outcome.

### Statistical Analysis

Results were expressed as means ± standard deviations. The paired Student's *t*-test, Mann-Whitney *U*-test, the Wilcoxon rank-sum test, and the Fischer exact test were carried out. Spearman correlation coefficients were considered when appropriate. For all statistical tests, a difference was defined as significant when the probability value was <0.05.

## Results

### Cerebral Blood Flow Velocity Measurements and PI

Preoperative MCA mean blood velocity varied from 8 to 143 cm/s. The average mean blood flow velocities in the MCA were 53 ± 38 and 51 ± 26 cm/s, respectively, in the most swollen cerebral hemisphere and in the opposite side (*p* = 0.88). The PI in the MCA ranged from 0.61 to 7.09. The average PI in the MCA was 1.85 ± 1.56 in the most swollen cerebral hemisphere and 1.73 ± 1.36 in the opposite hemisphere.

Postoperative MCA mean blood velocities varied widely from 39 to 155 cm/s. The average mean blood velocities in the MCA were 94 ± and 76 ± 16 cm/s, respectively, in the decompressed cerebral hemisphere and in the opposite side (*p* < 0.05). The PI in the MCA ranged from 0.46 to 1.30; the average PI in the MCA was 0.81 ± 0.18 in the decompressed cerebral hemisphere and 0.86 ± 0.22 in the contralateral hemisphere.

Following DC, mean blood velocities increased, on average, from 53 ± 38 to 94 ± 33 cm/s in the MCA of the decompressed cerebral hemisphere (*p* < 0.01), and from 51 ± 26 to 76 ± 16 cm/s on the contralateral side (*p* < 0.01), whereas PI decreased, on average, from 1.85 ± 1.56 to 0.81 ± 0.18 in the MCA of the decompressed cerebral hemisphere (*p* < 0.01), and from 1.73 ± 1.36 to 0.86 ± 0.22 on the contralateral side (*p* < 0.01) (Table 1).

**Table 1:** Cerebral blood flow velocity and pulsatility index before and after decompressive craniectomy.

**Variable**	**Side**	**Preop**	**Postop**	***P*-value**
Flow velocity	Decompressed	53 ± 38 cm/s	94 ± 33 cm/s	*p* < 0.01
Flow velocity	Opposite	51 ± 26 cm/s	76 ± 16 cm/s	*p* < 0.01
Pulsatility index	Decompressed	1.85 ± 1.56	0.81 ± 0.18	*p* < 0.01
Pulsatility index	Opposite	1.73 ± 1.36	0.86 ± 0.22	*p* < 0.01

### Classification of Patients and the Cerebral Hemispheres by Hemodynamic Patterns

Prior to DC, 10 patients (52.7%) presented cerebral oligoemia, 3 patients (15.8%) fulfilled the criteria for cerebral hyperemia, and 6 patients (31.5%) were assigned to have non-specific circulatory pattern (Table 2). Abnormal circulatory patterns were found in 13 patients (68.5%).

**Table 2:** Number of patients according to cerebral circulatory patterns before and after decompressive craniectomy.

**Circulatory patterns**	**Before craniectomy**	**After craniectomy**
Oligoemia	10 (52.7%)	1 (5.3%)
Hyperemia	3 (15.8%)	4 (21.0%)
Non-specific	6 (31.5%)	10 (52.6%)
Vasospasm	–	3 (15.8%)
Hyperemia / Vasospasm	–	1 (5.3%)
Total	19 (100%)	19 (100%)

Considering only the most swollen cerebral hemisphere, 10 patients (52.6%) had cerebral oligoemia, 7 patients (36.9%) presented non-specific hemodynamic pattern, and 2 patients (10.5%) had cerebral hyperemia. In the contralateral side (*N* = 17), 3 patients (17.6%) presented cerebral oligoemia, 13 patients (76.5%) showed non-specific hemodynamic pattern, and 1 patient (5.9%) had cerebral hyperemia (Table 3). Abnormal circulatory patterns were more frequent in the most swollen cerebral hemisphere than in the opposite hemisphere (respectively, 63.1 vs. 23.5%).

**Table 3:** Circulatory patterns in the most swollen cerebral hemisphere and in the opposite hemisphere before and after decompressive craniectomy.

	**Before surgery**		**After surgery**	
**Circulatory patterns**	**Most swollen hemisphere**	**Contralateral cerebral hemisphere**	**Decompressed cerebral hemisphere**	**Contralateral cerebral hemisphere**
Oligoemia	10 (52.6%)	3 (17.6%)	0	1 (5.9%)
Hyperemia	2 (10.5%)	1 (5.9%)	4 (21%)	1 (5.9%)
Non-specific	7 (36.9%)	13 (76.5%)	11 (58%)	15 (88.2%)
Vasospasm	0	0	4 (21%)	0
Total	19 patients (100%)	17 patients (100%)	19 patients (100%)	17 patients (100%)

After DC, 1 patient (5.3%) was found to present cerebral oligoemia, 4 patients (21%) fulfilled the criteria for cerebral hyperemia, and 3 patients (15.8%) were assigned to have cerebral vasospasm. One patient (5.3%) was classified as having hyperemia in one cerebral hemisphere and vasospasm in the contralateral hemisphere. Ten patients (52.6%) showed to have non-specific circulatory pattern (Table 2). Abnormal TCD-circulatory patterns were found in 9 patients (47.4%).

In the decompressed cerebral hemisphere, no patient had cerebral oligoemia, 11 patients (58%) presented non-specific hemodynamic pattern, 4 patients (21%) had cerebral hyperemia, and 4 patients (21%) were found to have cerebral vasospasm. In the contralateral side (*N* = 17), 1 patient (5.9%) presented cerebral oligoemia, 15 patients (88.2%) had non-specific hemodynamic pattern, and 1 patient (5.9%) showed to have cerebral hyperemia (Table 3). Abnormal circulatory patterns were more frequently encountered in the most swollen cerebral hemisphere than in the opposite hemisphere (42 vs. 11.8%, respectively).

### Clinical and Cerebral Hemodynamic Variables and Neurological Outcome

There was an inverse correlation between midline brain structures shift and GOS scores at 6 months post-injury (*r* = −0.46, *p* < 0.05). Also, the time interval from hospital admission to DC was inversely correlated with the degree of cerebral blood flow (CBF) velocity increase after surgical decompression (*r* = −0.50, *p* < 0.05) (Table 4). There was no correlation between postoperative cerebral circulatory responses and other clinical and imaging variables such as preoperative GCS score, GOS scores at 6 months post-injury, and neurological recovery based on favorable (good recovery and moderate disability) and unfavorable outcome (severe disability, vegetative state, or death) at 6 months follow-up.

**Table 4:** Correlation between neurological outcome and clinical variables and between postoperative cerebral hemodynamic changes and clinical variables^*^.

	**6-Months GOS**		**CBFV increase decompressed side**		**CBFV increase opposite side**	
	***P*-value**	**Correlation**	***P*-value**	**Correlation**	***P*-value**	**Correlation**
Age	0.319	−0.242	0.29	−0.256	0.556	−0.154
Sex	0.574	0.138	0.801	−0.062	0.802	−0.066
Interval Ad—DC	0.129	0.598	0.028	−0.503	0.03	−0.527
Preoperative GCS	0.113	0.375	0.301	0.251	0.472	0.187
Midline shift	0.044	−0.466	0.651	−0.111	0.27	0.284
CBFV increase decompressed side	0.113	0.645				
CBFV increase opposite side	0.158	0.545				

* *Interval Ad—DC, interval between hospital admission and decompressive craniectomy; GCS, Glasgow coma scale; CBFV, cerebral blood flow velocity; GOS, Glasgow outcome scale*.

## Discussion

### Role of Surgical Decompression

DC was proved to be effective for the treatment of cerebral oligoemia. Prior to surgery, more than a half of our patients (52.7%) had hemodynamic pattern of cerebral oligoemia (16) while only 5% of them (5.3%) after surgery. This finding can be explained by the decompressive effects of this surgery, which consist of reduction of ICP and increase of CPP, CBF, cerebral microvascular perfusion, and brain tissue oxygenation (1, 2, 12–15, 20). It is important to emphasize that these effects does not necessarily lead to cerebral hemodynamic improvement. In our cases, despite all these decompressive effects, almost half of the patients (47.4%) still had cerebral hemodynamic disturbances, potentially requiring postoperative cerebral hemodynamic monitoring, and possibly measures of cerebral hemodynamic control.

### Clinical Implications

Modern principles on CBF management in TBI patients recommend avoiding states of severe cerebral hyperemia and oligoemia (10, 16, 21). The former may result in cerebral blood volume rise, vasogenic edema enhancement, and the risk of intracerebral hemorrhage, while the latter may lead to cerebral ischemia and infarction. Both hemodynamic states can aggravate cerebral swelling and raised ICP. Therefore, the systemic and cerebral hemodynamic management should aim at adequate CBF, preferably coupled with metabolism, avoiding severe cerebral hyperemia, and oligoemia.

Methodically, causes of cerebral hyperemia (anemia, hypercapnia, arterial hypertension, hypervolemia, increased cardiac output, and cerebral metabolic crisis, drugs that induce microvascular dilation, etc.) must be investigated and treated if indicated. On the other hand, causes of cerebral oligoemia (hypocapnia, arterial hypotension, hypovolemia, dehydration, decreased cardiac output, raised ICP, drugs that induce microvascular constriction, among others) must be considered and corrected if possible. Factors that intensify cerebral metabolic activity (seizures and fever) must be avoided and treated, irrespective of the cerebral hemodynamic status, whether hyperemia or oligoemia, because such factors increase the energy requirement in the brain, worsening the uncoupling between cerebral blood flow and metabolism in cases of cerebral ischemia and/or the uncoupling between cerebral energetic need and brain energy production in cases of non-ischemic metabolic crisis due to mitochondrial dysfunction (22, 23). Cerebral oligoemia detected in our patients was not associated with significant arterial blood hypotension. During TCD examinations, factors that can cause low CBF velocity, such as suboptimal angle of insonation, arterial hypotension, arterial hypocapnia and intracranial hypertension must be considered and ruled out.

Recent papers disclosed that metabolic crisis in TBI patients undergoing DC cannot be explained only by cerebral ischemia (11, 13, 22–24). Some patients showed to have non-ischemic metabolic crisis characterized by impairment of oxidative phosphorylation in mitochondria, leading to failure of brain energy production. Therefore, there are hyperactivity of anaerobic metabolism pathway, failure of brain energy metabolism, and aggravation of cerebral swelling. The brain energetic failure associated with mitochondrial dysfunction triggers the cascade of free radical production, necrosis and apoptosis (11). A higher prevalence of mitochondrial dysfunction and ischemic episodes was reported in unfavorable outcome patients (11), reinforcing the importance of controlling both the cerebral hemodynamics and metabolism.

Non-ischemic metabolic crisis causes cerebral tissue acidosis due to anaerobic metabolism, despite high levels of tissue oxygen; as a consequence, microvascular paresis can occur leading to decrease in cerebrovascular resistance, impairment of cerebral autoregulation and hyperemia (12, 22, 23, 25). A recent review disclosed association between intracranial hypertension and dysfunction of cerebral autoregulation, which can persist after DC (26). The impairment of cerebral autoregulation can reduce the arterial blood pressure threshold needed to maintain suitable CBF (27).

Our results are important for guiding the intensive management of these patients. One patient from our series presented with hemodynamic pattern suggestive of cerebral hyperemia in one hemisphere and vasospasm in the other hemisphere. Therapeutic management of these patients may be challenging, chiefly if both hyperemia and vasospasm are severe. Arterial blood pressure augmentation therapy or surgical decompression for treating cerebral oligoemia may not be suitable for the hyperemic hemisphere; in contrast, measures for decreasing cardiac output may worsen ischemia in the oligoemic cerebral hemisphere. Such patients should be monitored closely with multimodal fashion to achieve a middle ground whereby correction of cerebral hypoperfusion does not cause significant worsening of contralateral cerebral hyperemia (28). It is worth stressing that traumatic intracranial expanding lesions and/or disturbed cerebrospinal fluid circulation contribute to the formation of pressure gradients in the intracranial space (29, 30), such as interhemispheric supratentorial pressure gradients (31), as well as to the hemispheric asymmetry of the pressure autoregulation (32) and critical closing pressure (33). Along with cerebral vasospasm, these pathophysiological events can explain, in part, our findings of cerebral hemodynamic heterogeneity, including heterogeneity between the cerebral hemispheres.

### Cerebral Hemodynamic Changes and Outcome

The data of the present study failed to show correlation between cerebral hemodynamics and clinical outcome. This does not mean that those correlations cannot exist. However, the statistical analysis revealed a significant inverse correlation between midline brain structures shift and GOS at 6 months after injury, suggesting that the greater the midline shift, the worse the clinical outcome. Also, a significant inverse correlation was found between the time interval from hospital admission to DC and cerebral hemodynamic changes after DC, indicating that the longer the time interval, the lower the degree of postoperative CBF velocity increases. Both facts can reinforce the idea that DC should be indicated early or, at least, should not be indicated too late.

### Limitations

This study has a number of caveats, mainly related to small sample size, possibility of type II error, and difficulties in obtaining serial TCD examinations and clinical data (from mechanical ventilation, sedation, vasopressors, intracranial pressure, cerebral metabolic, and electrical activity, neurological status, among others). Future studies should devise protocols that can investigate the temporal course of cerebral hemodynamics for each patient, and the impact of TCD results on guiding the treatment. Other limitations include the lack of data about the number of patients and the respective reasons for their exclusions during the recruitment process and the lack of sample size estimation in the planning of this research; the latter may limit the interpretation of the findings related to the correlation between cerebral hemodynamic patterns and clinical outcome. It should be noted that to date there are no data on this subject in the literature that can be used to calculate the sample size.

Concerning the MCA blood velocity threshold for vasospasm detection, flow velocities >140 cm/s can be more appropriate, however the higher the blood velocity, the lower the TCD sensitivity. Taking this into account, both flow velocities >100 cm/s and LR >3 were adopted in this study. The latter can improve the diagnostic discrimination between cerebral hyperemia and vasospasm.

Although little discussed, the diameter of MCA depends on factors such as blood pressure in the vessel lumen, intracranial pressure, and intrinsic vessel wall properties, among others. This means that the diameter of MCA may change following DC, making the interpretation of TCD results more difficult.

## Conclusion

DC leads to increase in CBF velocity and decrease in PI, indicating reduction in ICP. Our results showed a marked heterogeneity of postoperative cerebral hemodynamic findings among TBI patients with uncontrolled brain swelling who underwent DC, including hemodynamic heterogeneity between their cerebral hemispheres. DC was proved to be effective for the treatment of cerebral oligoemia. Not surprisingly, previous studies on TBI demonstrated cerebral heterogeneity in terms of circulation, pressure autoregulation, critical closing pressure, oxygenation, and metabolism (2, 16, 34). Our data reinforces the concept of heterogeneous nature of the TBI pathophysiology and suggest that DC as the sole treatment modality is insufficient. The combination of therapies (for instance, surgical decompression associated with the control of both CBF and metabolism) can potentially improve patients' outcomes. For the future, patients should be monitored in terms of ICP, cerebral hemodynamics and metabolism to allow individually planned treatments. In clinical practice, the identification of different cerebral hemodynamic and metabolic patterns and their significances may be useful for determining specific therapeutic strategies. TCD can be more used as a bedside monitoring method due to its low cost, non-invasiveness, wide availability, and relatively short time of examination. Unfortunately, this diagnostic tool depends on the operator skill to obtain and interpret the cerebral hemodynamic data, and does not directly quantify the CBF, but only its velocity. The finding of cerebral hemodynamic heterogeneity in severe TBI requires more TCD studies on this issue to have more practical clinical experience.

## Ethics Statement

This study was carried out in accordance with the recommendations of Hospital das Clinicas of the University of Sao Paulo Medical School research ethics committee (CAPPesq) with written informed consent from all subjects. All subjects gave written informed consent in accordance with the Declaration of Helsinki. The protocol was approved by the CAPP.

## Author Contributions

EB-S-S contributed conception and design of the study. EB-S-S, Md-L-O, RN, KA, EP, and FP collected the clinical data. EB-S-S wrote the first draft of the manuscript after discussions with Md-L-O, RN, KA, EP, and FP wrote some of the methods part. All authors contributed to manuscript revision, read, and approved the submitted version.

### Conflict of Interest Statement

The authors declare that the research was conducted in the absence of any commercial or financial relationships that could be construed as a potential conflict of interest.
